# Synthesis and Photocatalytic Properties of ZnWO_4_ Nanocrystals via a Fast Microwave-Assisted Method

**DOI:** 10.1155/2013/458106

**Published:** 2013-05-29

**Authors:** Jing Yan, Yanhua Shen, Feng Li, Taohai Li

**Affiliations:** Key Lab of Environment Friendly Chemistry and Application of Ministry of Education, College of Chemistry, Xiangtan University, Xiangtan 411105, China

## Abstract

High crystallinity of ZnWO_4_ nanoparticles has been successfully synthesized via a highly effective and environmentally friendly microwave route by controlling the reaction time and temperature. The products were characterized by X-ray powder diffraction (XRD), transmission electron microscopy (TEM), and Fourier infrared spectrum (FT-IR). The crystallinity was enhanced with the increase of the reaction temperature and time. The photocatalytic activities of ZnWO_4_ nanocrystals were evaluated by testing the photodegradation of rhodamine B (RhB) dye under ultraviolet (UV) light irradiation. The results indicated that as-prepared ZnWO_4_ was highly effective for the degradation of RhB. The degradation rate of RhB reached 98.01% after 6 h of UV illumination.

## 1. Introduction

Tungsten materials with novel architectures and physical and chemical properties are very useful for many potential applications such as flashing materials, LED [1], magnetic and fluorescent materials [2–8], optical fiber, humidity sensors [9], light emitting materials [10, 11], photocatalytic materials [12–18], scintillator [19], laser host [20], and nanoordered substrate materials [21, 22], so they are considered as an important class of functional materials. As an important photocatalyst, ZnWO_4_ has been applied for photocatalytic hydrogen production from water and mineralization of organic pollutants under UV light irradiation [18]. For the formation of tungstate nanomaterials with unique morphology and hierarchical organization, a great number of synthetic techniques, such as hydrothermal method [23, 24], microemulsion, and sol-gel method [25–28], have been developed in the past few years.

However, there are some shortcomings for the traditional synthetic methods: (1) the reaction temperature is too high; (2) the reaction cycle is too long; (3) the procedure is complex [23]. Obviously, the long reaction cycle will waste time. It is worse that the long reaction time may lead to the dendritic growth and increase the particle size. Chen and coworkers have synthesized ZnWO_4_ powder by hydrothermal method and demonstrating that particle aggregation is stimulated by temperature and time increase [2]. Wu et al. have successfully produced ZnWO_4_ photocatalyst via the sol-gel process in a temperature range of 450–800°C, but the grain size is difficult to be controlled [25]. Thus, the application of ZnWO_4_ photocatalyst has been limited, and an energy saving and environmently friendly synthesis method of nanoscale tungstate has received extensive attention. 

To date, as far as we know, the synthesis of ZnWO_4_ photocatalyst by microwave-assisted method is rare. Wu et al. have reported a microwave solvothermal route to synthesize ZnWO_4_ nanoparticles at 160°C. However, the route needed longer time (1–3 h) and organic solvent (ethylene glycol) [29]. Therefore, the development of fast and environmently friendly microwave-assisted methods for the synthesis of ZnWO_4_ nanocrystals is of great importance for broadening and improving their industrial applications. In this paper, we first successfully obtained high crystallinity ZnWO_4_ photocatalyst with a microwave-assisted process by several minutes and using water as solvent. Compared with the conventional methods, microwave irradiation is preferable due to its unique effects, such as volumetric heating, higher reaction rates, shorter reaction time, selectivity energy saving, and being environmentally friendly. During the degradation of rhodamine B (RhB) under UV light irradiation, the ZnWO_4_ exhibited high photocatalytic activities. The measurements suggested that photocatalytic property of the sample is related to the crystallinity, surface area, and dimension of particles. Moreover, the catalyst is relatively stable and can be reused.

## 2. Experimental Section

### 2.1. Synthesis

All syntheses were performed using a commercial multimode microwave synthesizer coupled with an automation system (Initiator 8 Exp). The reactions were conducted in 20 mL vessels. In a typical synthesis of the ZnWO_4_ powder, all the chemicals were of analytical grade and used without further purification. Sodium tungstate (Na_2_WO_4_·2H_2_O) and zinc chloride (ZnCl_2_) were the staring materials. Na_2_WO_4_·2H_2_O (0.14 g), ZnCl_2_ (0.13 g), H_2_O (10.0 mL), and poly(ethylene glycol) (0.12 g) were mixed. When the vessel was protected by a sealing cap, it was then transferred into the microwave cavity, and the temperature was increased rapidly to 180°C at a rate of 50°C/min. The reaction was performed at 180°C for several minutes, and the reaction vessel was quickly cooled to room temperature by a forced-N_2_ flow. The as-synthesized ZnWO_4_ nanocrystals were collected by centrifugation, washed with distilled water and absolute ethanol several times to remove impurities, and finally dried in vacuum at 80°C for 4 h.

### 2.2. Characterization

The morphology and microstructure of the sample were observed with a JEM-2010 transmission electron microscope operated at 120 kV. Further structural characterization was performed on FEI Tecnai F20 high-resolution field-emission transmission electron microscope (HRTEM) operated at 200 kV. XRD patterns of ZnWO_4_ photocatalysts were recorded by a MiniFlex II X-ray diffractometer operated at 40 kV and 40 mA using Cu K radiation (*λ* = 0.15406 nm). UV-Vis DRS was performed on a Hitachi U-3010, and BaSO_4_ was used as a reference. Fourier transform infrared spectra (FT-IR) were recorded on a Perkin-Elmer 1600 FT-IR spectrometer with a KBr disk. BET surface area was determined by nitrogen adsorption isotherm measure-ments at 77 K on a Micrometrics ASAP 2010. 

### 2.3. Measurements of Photocatalytic Activity

The photocatalytic activity of ZnWO_4_ catalysts was evaluated by degradation of RhB under a 300 W UV irradiation. ZnWO_4_ photocatalyst (25 mg) was dispersed in RhB solution (10 mg/L). Before UV irradiation, an adsorption-desorption equilibrium was established by ultrasonic and mechanical stirring for 30 min. After that, the solution was exposed to UV light irradiation under magnetic stirring. A little amount of reaction solution for UV-Vis spectroscopy analysis was taken from the photoreactor at appropriate time interval. 

## 3. Results and Discussion

**Figure 1:**
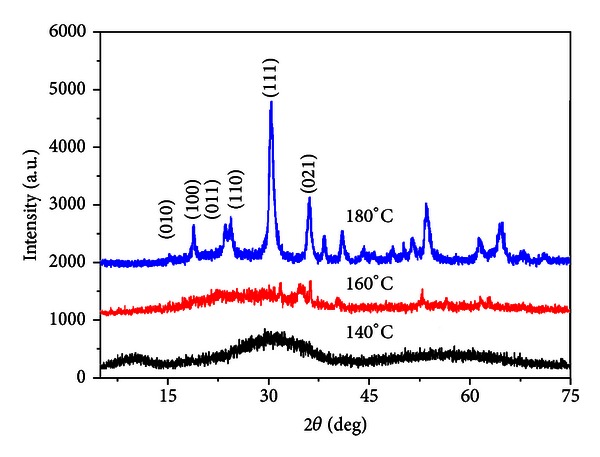
XRD patterns of ZnWO_4_ formed at a different reaction temperature for 5 min.

The phase composition of the samples prepared at different temperatures was analyzed by X-ray powder diffraction (XRD). As shown in Figure 1, there was no obvious ZnWO_4_ phase formation at 140 and 160°C. A sharpening of the peaks is observed at 180°C, and all peaks can be indexed in reference to pure ZnWO_4_, space group P2/c, and unit cell parameters consistent with those reported in JCPDS 15-0774. Therefore, it is reasonable to deduce that the increase of the microwave temperature improves the crystalline of ZnWO_4_ powder. Thus, the temperature increase stimulates effectively the crystallization of ZnWO_4_ nanoparticles.

**Figure 2:**
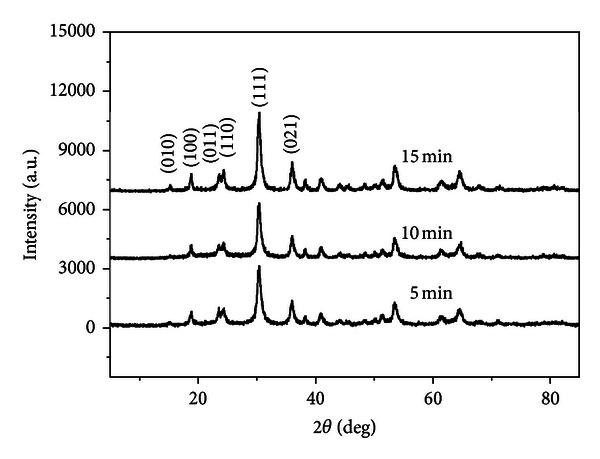
XRD patterns of ZnWO_4_ formed for a different reaction time at 180°C.

The growth process ZnWO_4_ nanocrystals was monitored by investigating the products obtained at different stages of the reaction using XRD techniques.  Figure 2 shows the XRD patterns of ZnWO_4_ samples irradiated at 180°C at different reaction stages. XRD patterns could be easily identified as a pure monoclinic wolframite structure ZnWO_4_ based on a JCPDS Card (no. 15-0774). The results indicated that the intensity of the diffraction peaks strengthens with the increase of reaction time, especially in the (010), (100), (011), (110), (111), and (021) crystal planes of ZnWO_4_. Small intensity (010) peak can be found in XRD patterns with 5 min reaction time. When the reaction time extended to 15 min, the (010) peak appeared. These results show that reaction time has an influence on the phase formation of ZnWO_4_. It also suggests that microwave method can achieve crystallization of samples in higher kinetics, which economized the energy and shortened the time.

**Figure 3:**
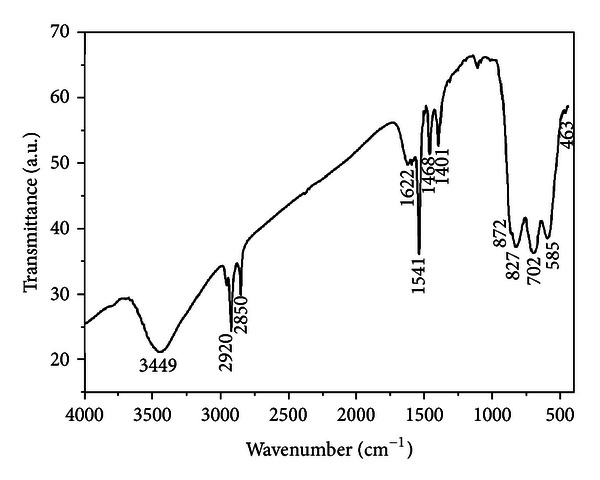
FT-IR spectra of ZnWO_4_ formed at 180°C for 15 min.

The FT-IR spectrum of ZnWO_4_ powders is shown in Figure 3. The bands at 463 cm^−1^ and 585 cm^−1^ are ascribed to the bending vibrations of W–O. The peaks at 702 cm^−1^ and 827 cm^−1^ are ascribed to the stretching vibrations of W–O. There exists a band at 872 cm^−1^ that arises from the bending and stretching vibrations of Zn–O–W [30]. The bands at 1401 cm^−1^, 1468 cm^−1^, 1541 cm^−1^, 1622 cm^−1^, and 3449 cm^−1^ are assigned to the O–H stretching and the H–O–H bending vibrations [31]. The weak bands at 2850 cm^−1^ and 2920 cm^−1^ are ascribed to the C–O vibration of CO_2_ in atmosphere [32]. The characteristic bands of the ZnWO_4_ indicated that the ZnWO_4_ formed.

**Figure 4:**
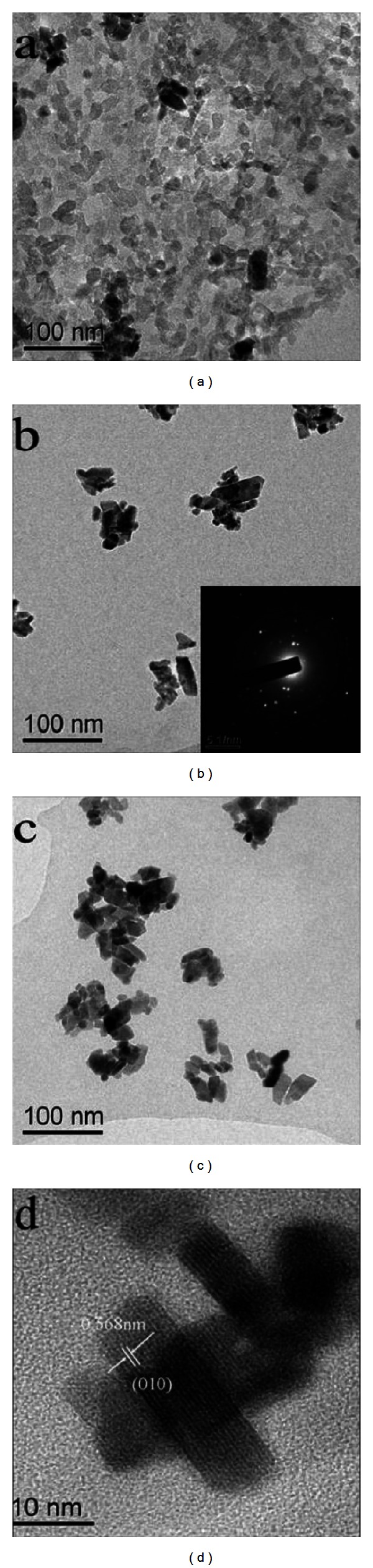
TEM morphologies of ZnWO_4_ at 180°C for different reaction times, 5 min (a), 10 min (b), 15 min (c), and HRTEM (d).

The TEM images of ZnWO_4_ catalysts prepared at 180°C for various times are shown in Figure 4. The ZnWO_4_ sample prepared at 180°C consists of the rodlike particles. The TEM images show that the length of ZnWO_4_ nanorods is about 26 nm and diameter is about 12 nm after 5 min of microwave irradiation. With the increase of irradiation time from 5 min to 15 min, the dimension of ZnWO_4_ nanorods increased. It can be seen that the reaction time plays a crucial role in diameter of the samples. The selected area electron diffraction (SAED) image recorded from the sample formed by 10 min microwave radiation indicates the single-crystal nature of the particles. This is verified by high-resolution field-emission TEM (HRTEM) image of ZnWO_4_ nanocrystals where *d*-spacing of about 0.586 nm is detected and well related to (010) interplane distance.

**Figure 5:**
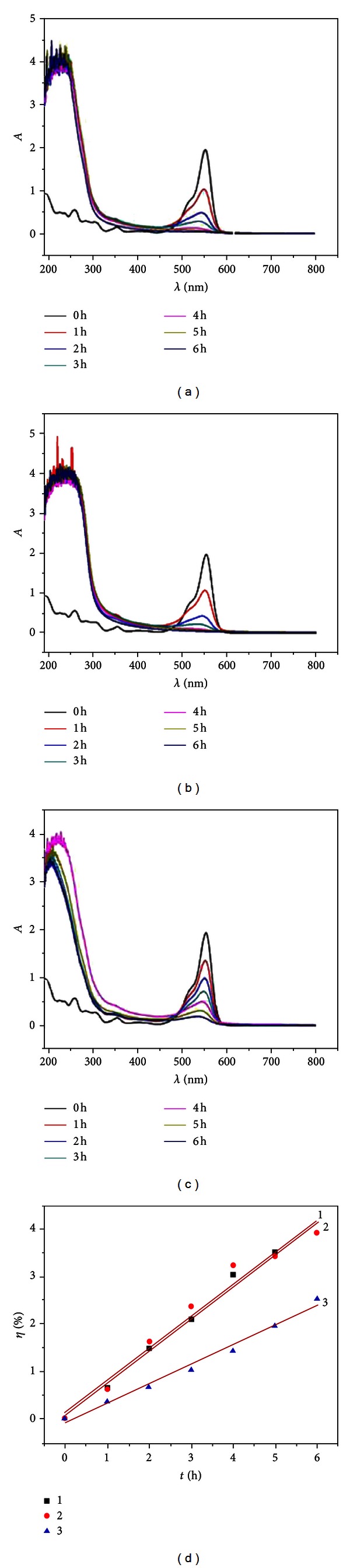
UV-visible absorption spectra changes of RhB with different as-prepared ZnWO_4_ samples: (a) 140°C, 5 min, (b) 160°C, 5 min, and (c) 180°C, 5 min. (d) Kinetics of degradation of RhB.

ZnWO_4_, as a semiconductor with a bandgap (*Eg*) around 3.9–4.4 eV, has high photocatalytic activity under UV light. The photocatalytic activities of as-prepared samples were tested via degradation of aqueous RhB under UV irradiation. The UV-visible absorption spectral changes taking place during the photodegradation of RhB for samples and photocatalytic kinetic curves of samples are shown in Figure 5, respectively. The results show that the sample prepared at 140°C has the highest photocatalytic activity, and after 6 hours of irradiation, the degradation rate of RhB can be nearly 98.01%. The kinetics curves of photodegradation of RhB are shown in Figure 5(d) where the curves 1, 2, and 3 are related to samples prepared at 140, 160, and 180°C, respectively.

 The linear relationship between *η* and time demonstrates that the photocatalytic degradation of RhB follows a pseudo-first-order kinetics:

(1) $$\begin{array}{c} \eta =\ln\left(\frac{C_{0}}{C}\right)=kt, \end{array}$$

where *C*_0_/*C* is normalized RhB concentration, *t* is the reaction time, and *k* is the reaction rate constant (min^−1^). The figure also shows that the degradation rate of samples prepared at 140°C is the best (curve 1), the reaction rate constant of which is calculated to be 0.6798 min^−1^. The degradation rate of samples prepared at 160°C is center (curve 2), the reaction rate constant of which is calculated to be 0.6760 min^−1^. The degradation rate of samples prepared at 180°C is the lowest (curve 3), the reaction rate constant of which is calculated to be 0.4133 min^−1^. The sample prepared at as high temperature as 180°C exhibits the lower ability to transform RhB compared with those prepared at 140–160°C. This indicates that the crystallinity structure of photocatalyst is the vital factor that affects the photocatalytic activity of materials.

**Figure 6:**
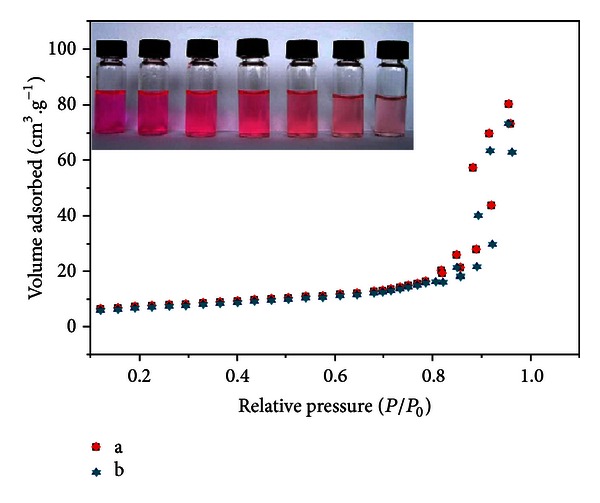
Typical nitrogen adsorption isotherms of as-prepared ZnWO_4_: (a) 140°C and (b) 160°C. Inset: Effect picture of photocatalytic degradation RhB by ZnWO_4_ prepared at 140°C for 5 min.

To obtain the information about the specific surface area of the as-prepared ZnWO_4_ samples, BET N_2_ adsorption analysis was performed. As shown in Figure 6, the nitrogen adsorption isotherm belongs to type II. The analysis showed that the specific surface areas of the samples prepared at 160°C and 140°C were 25.05 m^2^/g and 28.10 m^2^/g, respectively. The experimental results revealed that the specific surface area of samples prepared at 140°C is higher than the sample prepared at 160°C. Therefore, these demonstrations indicate that photocatalytic properties of ZnWO_4_ can be significantly improved by specific surface areas [33]. Figure 6 inset shows the photocatalytic effect of ZnWO_4_ prepared at 140°C for 5 min. We can see that the color of RhB gradually changes from violet red to shallow. This result coincided with the UV-Vis absorption spectra (Figure 5(a)). To evaluate further the photocatalytic activity, the final RhB solution was tested for the carbonation rate. The carbon content of nondegradated RhB solution was 6.445 m^2^/g, and the presence of carbon was not detected in the final RhB solution, which suggested that the dye almost completely was mineralized into CO_2_ and H_2_O. 

**Scheme 1:**
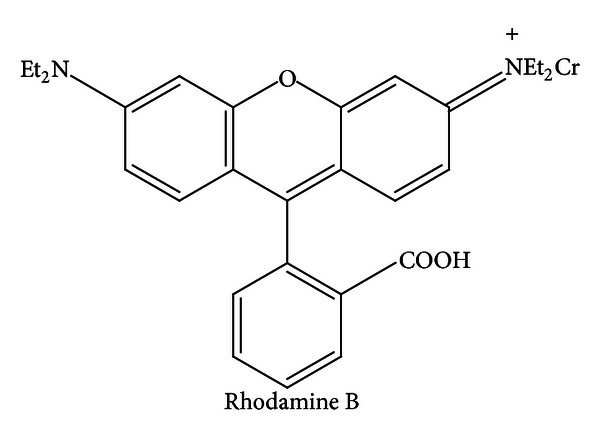
Chemical structure of RhB dye.

**Figure 7:**
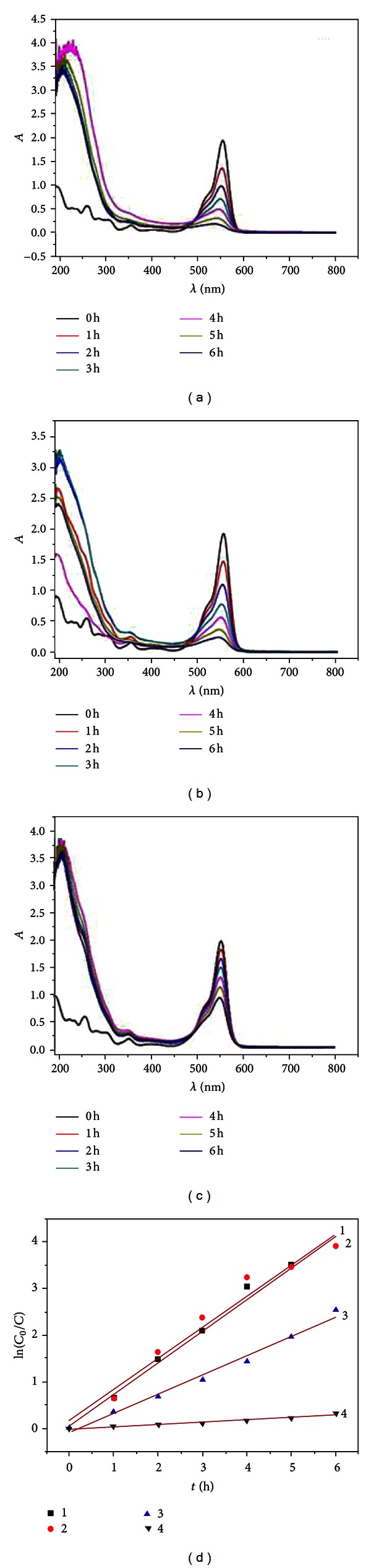
UV-visible absorption spectra changes of RhB with different as-prepared ZnWO_4_ samples at 180°C: (a) 5 min, (b) 10 min, and (c) 15 min. (d) Kinetics of degradation of RhB.

In this work, we also studied the photocatalytic degradation of ZnWO_4_ prepared at 180°C for different reaction times. The UV-visible absorption spectrum and dynamics curve of the aqueous solution of RhB with ZnWO_4_ under exposure to the ultraviolet light lamp for various durations are shown in Figure 7. The results show that the sample prepared at 180°C for 5 min has the highest photocatalytic activity, and after 6 hours of irradiation, the conversion of RhB can be nearly 92.12%.  Figure 7(d) is kinetics analysis of photodegradation of RhB, curves 1, 2, 3, and 4 represent photocatalytic kinetic curve of samples prepared at 180°C for 5, 10, and 15 min and no catalyst, respectively. The figure shows that the degradation rate of samples prepared for 5 min is the best (curve 1), the reaction rate constant of which is calculated to be 0.4133 min^−1^. The degradation rate of samples prepared by 10 min is intermediate, and the reaction rate constant is calculated to be 0.3762 min^−1^. The degradation rate of samples with reaction time of 15 min is the lowest (curve 2), the reaction rate constant of which is calculated to be 0.1261 min^−1^. Curve 4 shows almost no degradation of RhB after 6 hours of irradiation. When the reaction time is 5 min, samples have poor crystalline, small particles, and good dispersion, which lead to the higher photocatalytic activity. When the reaction time extended to 15 min, the sample has the best crystalline, large particle size, and large aspect ratio, but its photocatalytic activity is poorer than that of the sample prepared by 5 min [34]. This is consistent with previous conclusions. From the above analysis, we know that ZnWO_4_ nanopowders prepared at 180°C for 5 min have better photocatalytic activity, and its photocatalytic effect is shown in Scheme 1. Therefore, the crystallinity and particle size play important roles in photocatalytic activity of as-prepared ZnWO_4_.

**Figure 8:**
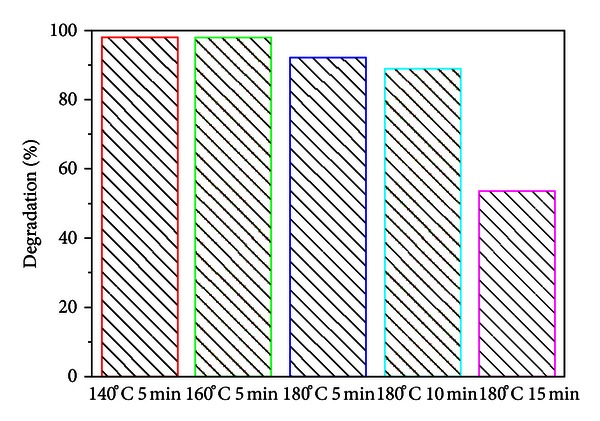
The activity of ZnWO_4_ for photodegradation of RhB under a different reaction condition.

Figure 8 compares the activity of ZnWO_4_ for photodegradation of RhB under a different reaction condition. It is observed that the ZnWO_4_ materials prepared at 140°C for 5 min have the maximal degradation rate. With higher reaction temperature, a decrease in photocatalytic activity is observed. These results can be explained by the poor crystallinity at low temperature, which leads to good catalytic activity. Also, with the extension of reaction time, the photocatalytic activity lets down which can be attributed to the large particle size.

**Scheme 2:**
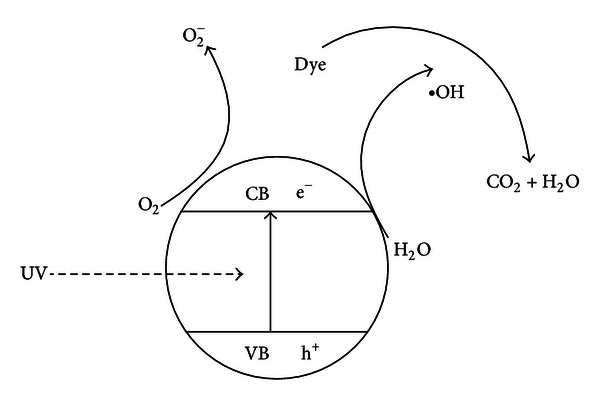
The photocatalytic mechanism of ZnWO_4_ under UV illumination.

On the basis of the above experimental observation, photocatalytic mechanism for ZnWO_4_ photocatalytic degradation RhB is speculated (Scheme 2). ZnWO_4_ energy band structure was composed by discrete, full-of-low-energy electron valence band (VB) and empty high-energy conduction band (CB); the valence band and conduction band are separated by the forbidden band. When the energy of absorption light is greater than the band gap, the valence electrons are stimulated to the conduction band, making the conduction band with charge, which has a reduction and the valence band resulting hole (h^+^) with oxidation. Simultaneously, the poor crystallinity leads to abundant lattice defects acting as holes [35]. These free electrons and holes migrate to the catalyst surface and react with adsorbed water, dissolved oxygen, which generate the high activity of hydroxyl radical (^•^OH) and superoxide anion (O_2_^−^), further react with organics in the dyes, and ultimately degraded to CO_2_ and H_2_O [36].

In addition, we repeated degradation of dye RhB three times with samples prepared at 180°C for 5 min to further study the stability of photocatalyst ZnWO_4_; the degradation rate remained at 90%. The experimental results showed that the catalyst was relatively stable and can be reused.

## 4. Conclusions

In this paper, the ZnWO_4_ nanoparticles were successfully synthesized by the microwave process. It is possible to control effectively the particle size and crystallization of ZnWO_4_ by adjusting the reaction temperature and reaction time. Comparing the photocatalytic property of ZnWO_4_ prepared at a different reaction time and temperature, we conclude that photocatalytic property of the sample is related to the crystallinity, surface area, and dimension of particles. The photocatalytic activity of samples prepared at reaction temperature of 140°C and reaction time of 5 min is the best. Furthermore, the ZnWO_4_ powder products showed stable photocatalytic activity for the degradation of RhB under UV light irradiation.
